# Selecting Valid Correlation Areas for Automated Bullet Identification System Based on Striation Detection

**DOI:** 10.6028/jres.116.010

**Published:** 2011-06-01

**Authors:** Wei Chu, John Song, Theodore V. Vorburger, Robert Thompson, Richard Silver

**Affiliations:** National Institute of Standards and Technology, Gaithersburg, MD 20899

**Keywords:** edge detection, firearms identification, morphology, topography, striation mark

## Abstract

Some automated bullet identification systems calculate a correlation score between two land impressions to measure their similarity. When extracting a compressed profile from the land impression of a fired bullet, inclusion of areas that do not contain valid individual striation information may lead to sub-optimal extraction and therefore may deteriorate the correlation result. In this paper, an edge detection algorithm and selection process are used together to locate the edge points of all tool-mark features and filter out those not corresponding to striation marks. Edge points of the resulting striation marks are reserved and expanded to generate a mask image. By imposing the mask image on the topography image, the weakly striated area(s) are removed from the expressed profile extraction. Using this method, 48 bullets fired from 12 gun barrels of six manufacturers resulted in a higher matching rate than previous studies.

## 1. Introduction

Firearms often leave unique microscopic striation marks on fired bullets. The analysis and comparison of these features play an important role in forensic firearm evidence analysis in crime investigation. Since the 1990s, automated firearm identification systems have received considerable attention and application [1–7]. An automated firearms identification system ranks the stored images according to certain similarity metrics to a subject bullet. The ranking of the bullet images in a database gives the firearms examiner an ordered list of the most promising potential matches for further microscopic comparison. The comparison might determine whether any listed bullet images match the subject bullet.

In some studies, the cross correlation function of feature profiles has been adopted as the similarity metric of two land impressions (also called land engraved areas, LEAs) [2, 3, 6, 7]. When the compressed profile is extracted from a land impression by averaging multiple surface profiles measured perpendicular to the striation direction, invalid areas may be encountered that are weakly striated and do not contain valid striation information. The inclusion of these areas may lead to sub-optimal extraction of the feature profile and subsequent deterioration of correlation results. In our previous study [8], an automated method was developed to select correlation areas with good striation quality. But a limitation of the program is that it could only be used for selecting a rectangular area along the longitudinal (bullet axis) direction. Invalid areas could not be removed from the rectangular area for further signature profile extraction and correlation.

In this paper, a method is proposed for selecting the valid correlation area and for excluding the embedded invalid areas within the land impression. Valid striation marks are located by applying an edge detection technique and by subsequent filtering processes so that only valid correlation areas are used for the compressed profile generation. This method for automatically removing invalid areas was tested on topography data from 48 bullets that were fired from twelve gun barrels of six manufacturers. With the invalid areas removed, the correlation of compressed profiles extracted from the remaining good areas achieved a higher matching rate than our previous study results.

## 2. Method

### 2.1 Image Preprocessing

**Fig. 1:**
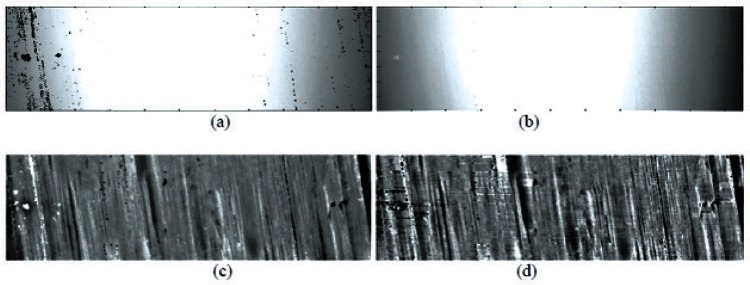
Preliminary processing results for a bullet land impression image: (a) raw data; (b) after removal of dropouts and outliers and interpolation; (c) after Gaussian filter; (d) after top-hat transform. The bullet nose is toward the top of the page.

Besides individual characteristics which are used for forensic bullet identification, the raw data of a land impression also includes components of bullet curvature, form error, noise, outliers or other unreliable data points. Therefore, preliminary image processing must be performed to remove or attenuate these components. In this study, 3D topography images were acquired by a commercial confocal microscope. The preliminary image processing consists of three steps: 1) identify and remove dropouts (points that the sensor was not able to acquire) and outliers (points that the sensor managed to acquire, but which are inaccurate or noisy), and replace these points with interpolated data; 2) apply a bandpass Gaussian filter to remove low frequency curvature, form error and high frequency noise; 3) perform a morphological top-hat transform to detect subtle individual structures and remove any remnant curvature on the bullet surface not removed by Gaussian filtering. Figure 1 shows an example of a 3D bullet topography image with its sequentially processed results.

### 2.2 Locating Striation Marks

An edge detection technique is used to find and locate valid striation marks. The word “edge” itself refers to physical properties, such as boundaries, of objects. But edge points in an image may have various meanings. In image processing or computer vision, edge detection is a process that primarily measures, detects, and localizes changes of intensity in the image [9]. It has the desirable property of drastically reducing the amount of information to be processed while preserving information about the shapes of objects in a scene [10,11]. Edges in image analysis do not necessarily correspond to the boundaries of an object, and can be specified in terms of texture properties in images that do not contain discrete objects [12]. In an image of a fired bullet surface, the primary textures that concern firearms examiners are striation marks engraved by the gun barrel. Applying the edge detection technique, the positions of these striation marks can be located. There are many methods to perform edge detection. For this research, the Canny edge detector was selected due to its merits of low error rate and good localization of edge points. The Canny edge detector is a multi-stage algorithm that detects a wide range of edges in images. On the basis of other traditional edge detectors that calculate first or second order derivatives of an image, Canny designed a detector to achieve optimal edge detection by using non-maximum suppression and hysteresis thresholding [10].

**Fig. 2:**
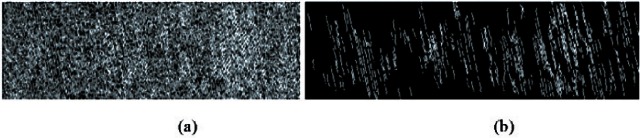
Edge detection and analysis result: (a) initial edge detection result from flattened image as Fig. 1(d) using Canny detector; (b) ultimate image of striation marks using edge filtering.

Figure 2 shows an example of edge detection. Figure 2(a) is a primary edge detection result calculated from Fig. 1(d), the flattened land impression topography image, using the Canny detector. It highlights all detected feature edges on the bullet surface, including both useful features associated with the striation marks impressed by the barrel, and useless minor features located within the image area. Therefore, a filtering process is further applied to the primary edge detection image to distinguish the striation edge points from the irrelevant edge points. The striation marks that concern firearms examiners are formed when the bullet is forced through the gun barrel. These striation marks are approximately parallel and straight, and they have a continuous span in a direction similar to the rifling tilt (twist) angle. Although the edge detection results originating from other features irrelevant to striations may have various shapes, they do not have the characteristics described above. Because of these differences, they can be filtered out from the primary edge image. We define a connected collection of edge points separated from other collections as a connection unit. The vertical span of each connection unit in Fig. 2(a) is checked and compared with a threshold length *L*. Any such unit, whose vertical pixel position difference between its top point and bottom point is greater than the threshold *L*, is reserved. Otherwise, it is removed. Stricter selection criteria can be applied by checking whether the tilt angle or degree of straightness for each connection unit is within a specifiable tolerance. Figure 2(b) shows the processed striation image after the edge filtering process.

### 2.3 Invalid Area Removal and Generation of the Compressed Profile

**Fig. 3:**
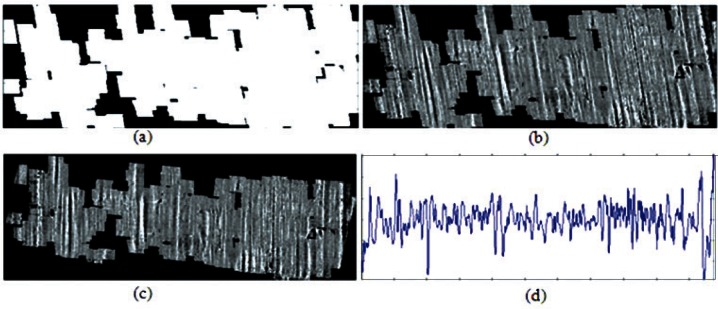
Generation of feature profile: (a) mask image; (b) topography image with invalid areas removed; (c) rotated image of (b); (d) compressed profile.

The purpose of using edge detection is to locate and select the valid correlation area for generating the compressed profile for further calculation of correlation scores. In a striation edge image, those edge points composing one connection unit only represent the position where intensities change most severely in a single striation mark. The entire local area in a topography image near each striation mark needs to be included in the subsequent analysis. Therefore a broadening of each striation mark is performed next, and the broadened image is used as a mask image since in this binary image the areas that contain valid striation marks are distinguished from those that do not. So the invalid areas are excluded from generating the compressed profile. Figure 3 shows this procedure. A one dimensional structuring element expands each edge point of Fig. 2(b) horizontally without change in the vertical direction. The length of the structuring element is decided from the average width of the striation marks. The number and width of striations in a land impression can vary depending on the size of the bullet and the characteristics of the barrel. For example, according to Biasotti’s study, the Smith and Wesson barrel with a land impression width of 2.4 mm has an average of 60 striations with total striation counts between 16 and 97 [13]. Observing the land impression images used in our study, 50 pixels (about 0.08 mm) is a reasonable choice for the average width of a structuring element. Figure 3(a) is the broadened result of Fig. 2(b), the striation edge image. By imposing it as a mask on Fig. 1(d), the flattened topography image, Fig. 3(b) is obtained with invalid areas removed. Figure 3(c) is an image rotated from 3(b) by the twist angle so that all striations are vertical. Projecting and averaging all valid data points vertically for each pixel column, the generated compressed profile is plotted as 3(d).

## 3. Experiment and Result

### 3.1 Experimental Data and Correlation Calculation

In order to test the selection method for the valid correlation area based on edge detection, two 9 mm caliber Luger gun barrels from each of six brands —Beretta, Ruger, Taurus, Browning, Sig Sauer and Bryco—were selected for the experiment. Two brands of bullets, and two bullets of each brand, were fired from each barrel. Therefore, topography images were measured and correlated from a total of 48 bullets (4 bullets 2 barrels 6 firearm brand). Assuming that the bullet images can provide enough class characteristic information (LEA width, for example) to distinguish bullets fired from their own barrel models, each bullet was correlated only with the other seven bullets fired from the same brand of barrel.

Since each bullet fired from these gun models has six land impressions, a pair of bullets was correlated at six possible phases. Let Bullet 1(B1) and Bullet 2(B2) indicate a pair of bullets to be correlated. Each land impression of B1 is correlated with each of B2, resulting in a 6 by 6 matrix with 36 cross correlation values. If bullets B1 and B2 were fired from the same gun, there would be one phase orientation where each land impression of B1 should be highly correlated with each land impression of B2 in succession. The customary correlation metric, sometimes called the “max phase” [1], is calculated as the sum of the cross-correlation scores for six land impressions at the optimum phase orientation where that sum score is highest. For each bullet, all seven other bullets fired from the same brand barrels will be ranked in order of their “max phase” scores.

### 3.2 Result and Comparison

For the evaluation, two different parameters are calculated and listed in Table 1 and compared with values calculated from our former study which did not contain a procedure to automatically exclude the invalid areas individually [8]. In the table, experiment 1 represents the former experiment; while experiment 2 represents the correlation calculation using the methodology described above. The two parameters, *N* and *p*, are described below.

#### *N*—total number that are correct matches

For each group of bullets fired from same brand gun barrel, *N* represents the total number of correct matches occupying the top 3 positions in 8 ranking lists. In an ideal condition, all three bullets fired from the same barrel should have the highest “max phase” scores and occupy the top three positions in a ranked list. Therefore, the maximum possible value for *N* should be 8 × 3 = 24 and the statistical expectation value for a random condition is 3/7 × 3 × 8, approximately equal to 10.3.

#### *p*—overlap metric

An overlap metric *p*, discussed earlier in Ref. [14], is calculated here in order to characterize the quality of the separation (or the degree of overlap) between distributions of matching and non-matching correlation scores. The distributions are calculated by fitting Gaussian distributions to the histograms of correlation values. The overlap metric describes the probability that the cross correlation value of a randomly chosen member of the non-matching distribution is larger than the cross correlation value of a randomly chosen member from the matching distribution. The parameter *p* quantifies the potential for making accurate matches. The smaller the value of *p* the better the separation between matching and non-matching distributions. Zero overlap metric *p* implies ideal identification without error. If the two distributions were the same, then *p* would be 0.5. For each type of firearm in this experiment, a distribution was fitted to the max phase scores for 32 non-matching bullet pairs and a distribution was fitted to the max phase scores for 24 matching bullet pairs.

From Table 1, we can see that the statistical result of six groups of bullets have improved from experiment 1 to experiment 2, except for the SIG group which exhibited a slight statistical deterioration. Further analysis for this group of bullets will be described in the next section. The total correct matching number of 48 bullets improved from 102 to 111 with an 8.8 % improvement. If the bullets of SIG group are not considered, the improvement is calculated to be more than 10 %.

## 4. Discussion

Striation marks are the foundation for bullet signature correlation and they also determine the reliability of bullet identification. The greater the quantity of clear striation marks a bullet image possesses, the higher the calculated striation density will be. In our previous work [15], the method of extracting striation edges is given in detail and a primary conclusion is that the striation density has strong correlation with identification accuracy. A striation density parameter, *d_s_*, which is defined as the ratio of the number of pixels on the striations to the total number of pixels in the area, can provide examiners with a means to estimate the quality of bullet images and therefore predict the potential for identification. In this paper, a method of valid correlation area selection based on striation edge detection is developed and correlation results further support this hypothesis. See the statistical results for all 48 bullets listed in Table 2. The average *d_s_* ’s for each group of bullets are obtained from 6 land impressions × 8 bullets for a total 48 land impression images. It can be seen that bullets with higher striation density are more likely to give a higher identification rate.

The threshold length *L* has a significant effect on distinguishing a valid striation area from an invalid area, and it also affects the calculation of *d_s_*. Too short a threshold length does not effectively remove the edge points of features irrelevant to striation marks. Too long a threshold length excessively removes some valid topography information. Either case could decrease the identification accuracy compared with an optimal *L*.

**Fig. 4:**
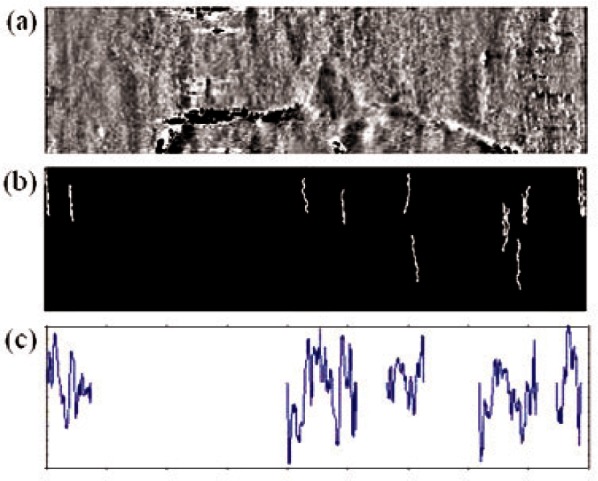
(a) A grayscale land impression image of a bullet fired from a SIG barrel; (b) the striation edge image filtered using 30 pixels threshold length; (c) generated compressed profile.

In the experiment, several different threshold length *L*’s are tried. Based on the statistical correlation result for all 48 bullets, the threshold is chosen to be 30 pixels (or about 47 μm) on the LEA surface. However, the 30-pixel threshold may not be optimal for all bullets. By dividing bullets of the SIG group into two sub-groups with four bullets each corresponding to the two different barrels they are fired from, the average *d*_s_ and *N* values obtained are 0.9 %, 2.12 % and 4, 11, respectively. The first barrel engraves poor striation marks on bullets fired from it. When a threshold of 30 pixels is selected, only a few edge points can be included; hence, the compressed profile contains a large number of empty points. Figure 4 represents such an example, where loss of too much topography information and correlation using a compressed profile having too few data points leads to a poor correlation result.

**Fig. 5:**
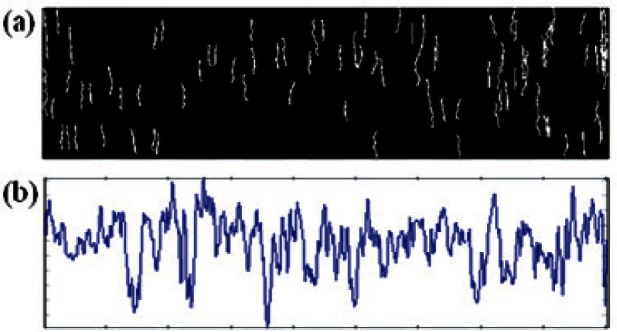
(a) Striation edge image filtered using 20 pixels threshold; (b) generated compressed profile.

Better results can be obtained for this barrel if a smaller threshold length *L* is applied to the 24 land impression images of the four bullets in this sub-group. Reducing *L* to 20 pixels, the revised striation image and the compressed profile for the data of Fig. 4(a) are shown in Fig. 5. Recalculating the correlation scores of the bullets of the SIG group, the correct matching number *N* for 4 bullets fired from the former barrel increases from 4 to 7. For the 8 bullets of the whole SIG group, the values of *N* and *p* correspondingly improved to 18 and 0.20, respectively.

Therefore, a conclusion may be drawn that a reasonable threshold length *L* may vary from one barrel to the next. At present, the selection of the value of *L* still depends on an empirical method. A more intelligent method to select the threshold according to the individual characteristics of bullets could further improve the matching accuracy for bullet topography.

## Figures and Tables

**Table 1:** Correlation result comparison between two sets of experiments

		Taurus	Bryco	Ruger	SIG	Browning	Beretta
Experiment 1	*N*	14	9	24	16	15	24
	*p*	0.277	0.554	0.006	0.217	0.271	0.026
Experiment 2	*N*	18	13	24	15	17	24
	*p*	0.260	0.418	0.003	0.248	0.230	0.018

**Table 2:** Statistics of average striation density and total correct matching number

	Taurus	Bryco	Ruger	SIG	Browning	Beretta
Average *d*_s_ (%)	3.66	1.41	3.9	1.51	2.23	6.86
*N*	18	13	24	15	17	24
